# High Dielectric Performance of Polyamide 66/Poly(Vinylidene Fluoride) Flexible Blends Induced by Interfacial Copolymer for Capacitors

**DOI:** 10.3390/polym8010002

**Published:** 2015-12-24

**Authors:** Rui Li, Zixuan Chen, Jianzhong Pei

**Affiliations:** Highway School, Chang’an University, Xi’an 710064, China; lirui@chd.edu.cn (R.L.); echoslim@163.com (Z.C.)

**Keywords:** compatilbilizer, blend, dielectric property, interaction, all-polymer

## Abstract

The copolymer VAMA was synthesized from vinyl acetic and maleic anhydride. A new all-polymeric blend with a high dielectric constant (ε) has been developed by blending polyvinylidene fluoride (PVDF) with vinyl acetic-maleic anhydride modified polyamide (PA66-*g*-VM). The blend shows high dielectric constants (ε_blend_ = 20) and excellent mechanical properties. The SEM investigations suggest that the enhanced dielectric behavior originates from significant interfacial interactions between polymers. The XRD demonstrates that the compatibilizer affects the crystalline behavior of each component. Furthermore, the stable dielectric constants of the all-polymeric blends can be tuned by adjusting the content of the compatibilizer. The created high-ε all-polymeric blends represent a novel type of material that is technologically simple, easy to process, and of a relatively high dielectric constant, with application for flexible electronics.

## 1. Introduction

With the continuous development of science and technology, requirements become higher to the energy efficiency of materials, and it is hard for single polymer to meet requirements [1,2]. The polarization mechanism of polymer tells us that the structure, crystal form, and crystalline of polymer have great influence on the dielectric constants of polymer. Thus, two or more polymers blended to prepare polymer blending material make it possible to develop polymer film energy storage material. Polymer-based blend is combined with the respective advantages of two polymer materials. It has the advantages of processing convenience, fine flexibility, and the ability to take any shape [3,4]. It has extensive application prospects to blend different polymer matrixes in electronic systems. Especially, its applicability in preparing electrical appliance materials which can be used in modern electronic industry, such as the preparation of embedded capacitors, *etc.* [5,6,7].

According to the property index requirements of capacitor material, the paper selected two typical polar polymers which are polyvinylidene fluoride (PVDF) and polyamide (PA) to blend. In the previous study, the mechanical-electrical properties and improvement method were reported about PA11/PVDF blend system [8,9,10]. However, in the actual application, the expensive price of PA11 limited applications in many fields. As a nylon material which is frequently used, PA66 and its inherent excellent properties were focused on for a long time. However, there are still few studies about PA66/PVDF blend energy storage materials. As we know, the melting point of PA66 is 220–240 °C, but the processing temperature of PVDF is 160–180 °C. The melting point of PA66 was lowered by grafting process, thus the mechanical melt blending problem of PVDF and PA66 can be solved. In this chapter, the copolymer VM from acetic acid vinyl ester and maleic anhydride were synthesized. Next, the grafted copolymer VM-*g*-PA66 from PA66 and VM were prepared by melt blending graft. Grafting reaction can lower the melting point of PA66, and the blends of both can be obtained from VM-*g*-PA66 and PVDF by melt blending through torque rheometer, studying the mechanical properties and morphology of blends. Thus, this paper is going to test microstructure characterization on the electrical properties so as to discuss the relationship of polyamide structure and electrical properties from the changes of molecular chain structure and the position of amide groups to further improve the electrical properties of material.

## 2. Experimental

### 2.1. Materials

PVDF (*T*_m_ = 175 °C, ρ = 1.75 g/cm^3^) and PA66 (*T*_m_ = 225 °C, ρ = 1.15 g/cm^3^) powders were supplied by the Shanghai 3F Corporation (Shanghai, China) and the Sinopec Baling Branch Corporation (Changsha, China), respectively. The related products are commercially available.

### 2.2. Synthesis of Copolymer of Vinyl Acetate and Maleic Anhydride

Methylbenzene (200 mL) was added to a three-mouth flask equipped with a stirrer, thermometer, and reflux condensing tube, and then 0.10 mol powdered maleic anhydride was added and dissolved into methylbenzene slowly after stirring and heating. After the solution turns to be transparent, 0.10 mol acetic acid vinyl ester and 1.4% initiator azodiisobutyronitrile were added. The temperature was controlled at 80 °C. After the copolymer subsided, the reactant was obtained by cooling. After filtering and washing with dichloroethane five times, the unreacted maleic anhydride and initiator were removed. After vacuum drying under the circumstance of 60 °C, white powdered copolymer was obtained. This paper abbreviates the copolymerization of VAc and MAH to VM from now on.

### 2.3. Preparation of VM-g-PA66 and VM-g-PA66/PVDF

**Figure 1:**
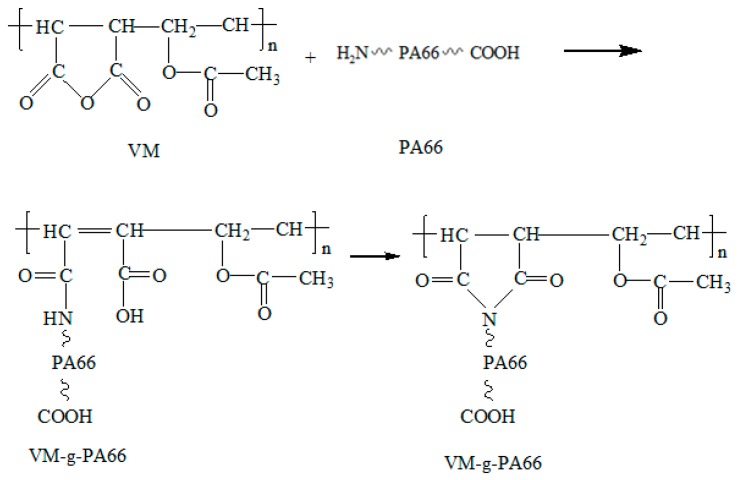
Reaction scheme of PA66 and VM copolymerization.

PA66 was dried for 24 h at 100 °C in drying oven. VM copolymer was placed for 8 h with vacuum drying at 60 °C. The PA66 was added to torque rheometer with 220 °C. The prepared and dried VM copolymer was added after PA66 was melted completely. After mixing the blends for a while, the material can be taken out and cooled to have standby application. See the grafting reaction process of PA66 and VM in Figure 1. PA66, VM-*g*-PA66 and PVDF were mixed for 20 min based on different volume ratios in an XSS-300 torque rheometer (Shanghai Kechuang Rubber Plastic Machinery Co., Ltd, Shanghai, China), and then a press vulcanizer was used to press the evenly pressed VM-*g*-PA66/PVDF blends into thin a blended sheet which was 2 mm thick. In addition, a micro tablet machine was used to press sheets into around 1 millimeter of thickness. Finally, the samples were polished and silvered with silver paint.

### 2.4. Instrumental Analysis

The dielectric properties of the samples were performed by HIOKI3532-50 LCR, the frequency was from 10^2^ to 5 × 10^6^ Hz at room temperature. The microstructures of the blends were characterized by using X-ray diffraction (XRD, Japanese Rigaku Company, Tokyo, Japan), differential scanning calorimetry (DSC, PerkinElmer Apparatus Co., Ltd, Berlin, German), Scanning Electron Microscopy (SEM, Japanese Electronics Corporation, Tokyo, Japan), and Fourier Transform Infrared Spectroscopy (FTIR, German Bruker Company, Billerica, MA, USA). Dynamic mechanical properties were measured using a dynamic mechanical thermal analyzer (DMTA, Shenzhen Xinsansi Electronics Corporation, Shenzhen, China) at 1 Hz and at a heating rate of 3 °C/min from −100 to 150 °C.

## 3. Results and Discussions

### 3.1. Structure Analysis

**Figure 2:**
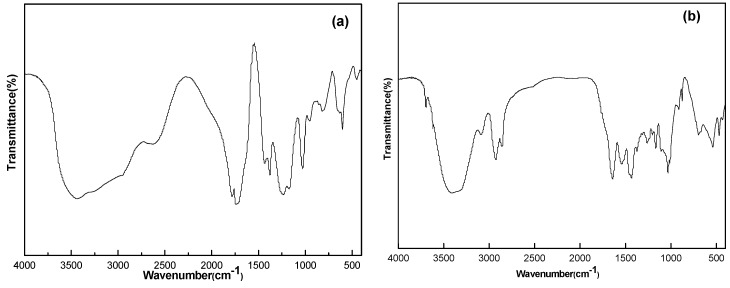
FT-IR spectrum of copolymer (**a**) VM; and (**b**) VM-*g*-PA66.

The infrared spectrum of acetic acid vinyl ester and maleic anhydride copolymer is tested by Fourier transformation infrared spectrometric analyzer. It can be seen from the Figure 2 that the absorption peak of wave number is 1780 cm^−1^ for carboxylic acid C=O stretching vibration and 1231 cm^−1^ for C–O stretching vibration; they are the characteristic structures of acid anhydride. The wave number is 1780 cm^−1^ for carboxyl ester C=O stretching vibration and 1374 cm^−1^ for methyl groups C–H flexural vibration. The intensity of the absorption band in wave number 1374 cm^−1^ is greater than in 1433 cm^−1^, which is an obvious characteristic of vinyl acetate structure [11,12]. Thereby, it proves the reaction of copolymerization between acetic acid vinyl ester and maleic anhydride. See the reaction equation of VM in Figure 2.

See the infrared spectroscopic analysis of grafted copolymer in Figure 2b. The peaks of 1650 and 1540 cm^−1^ are corresponding to the absorption peak of C=O and the absorption peak of N–H and C–N of amide. There was a large and wide peak envelope in the range of 2700–3600 cm^−1^. This is the characteristic peak of the carboxyl group. However, 1433 and 1374 cm^−1^ are the characteristic absorption peaks of vinyl acetate, which proves that PA66 and VM can react to grafted copolymer by melt blending.

**Figure 3:**
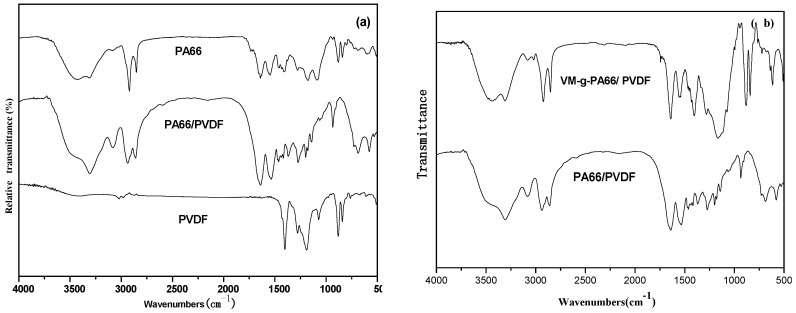
FT-IR spectrum of PA66/PVDF blends: (**a**) without VM; (**b**) with VM.

Figure 3a is the FT-IR spectrum of PA66/PVDF blends. It can be seen from the figure that PA66 has a significant absorption peak at 3301 cm^−1^, which is corresponding to the stretching vibration of N–H. The strong amide I band at 1640 cm^−1^ is produced by the C=O stretching vibration of amide group. The characteristic absorption peaks of amide at 1555 and 1405 cm^−1^ were produced by C–N stretching vibration and N–H flexural vibration. For PA66 and PVDF, FT-IR spectrum of PA66/PVDF blend film has no new peak or significant deviation. It means there is no bulking agent, PA and PVDF cannot have chemical reaction by melt blending.

Figure 3b is the infrared spectrum of PA66/PVDF and VM-*g*-PA66/PVDF. When the infrared spectral curve of VM-*g*-PA66/PVDF was compared with that of VM, it can be found that the two acid anhydride peaks disappear at 1780 and 1740 cm^−1^. When the infrared spectral curve of VM-*g*-PA66/PVDF is compared with it of VM, it can be found that VM-*g*-PA66/PVDF characteristic peak of cyclic amide disappears at 1414 cm^−1^, which shows the anhydride of VM molecule has chemical reaction with –NH of PA66 molecule.

**Figure 4:**
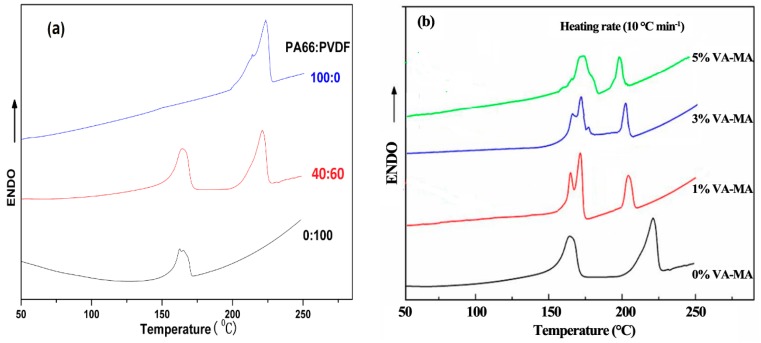
DSC thermograms of PA66/PVDF blends at 10 °C·min^−1^: (**a**) without VM; (**b**) with VM.

Figure 4 is DSC curve of PA66/PVDF blends. It can be seen from the figure that the melting peak position of PA66 is at 223.2 °C. The melting peak of PA66/PVDF blends were coexisting after blending PA66 and PVDF, it shows PA66 and PVDF of PA66/PVDF blends can also be crystallized.

**Figure 5:**
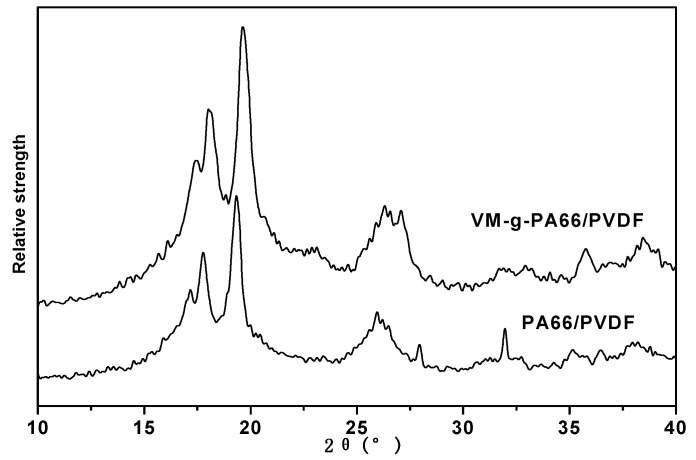
XRD pattern of PA66/PVDF blends.

Figure 5 is XRD pattern of PA66 and PVDF blends. It can be seen from the figure that there are the α crystal-form diffraction peak of pure PA66 monomer at 2θ = 20.3° and β crystal-form diffraction peak at 2θ = 22.8°. It can be seen by blending PVDF and PA66 that the α crystal-form diffraction peak of PA66 monomer at 2θ = 20.3° was weakened with the accession of PVDF, but the β crystal-form diffraction peak at 2θ = 22.8° was enhanced, which shows that PVDF can inhibit the formation of α crystal form of PA66 and promote the β crystal of PA66. Besides, there are α crystals formed of PVDF in PVDF/PA66 blends, which shows that the accession of PA66 cannot promote to form β crystal or inhibit the formation of α crystal form in PVDF.

**Figure 6:**
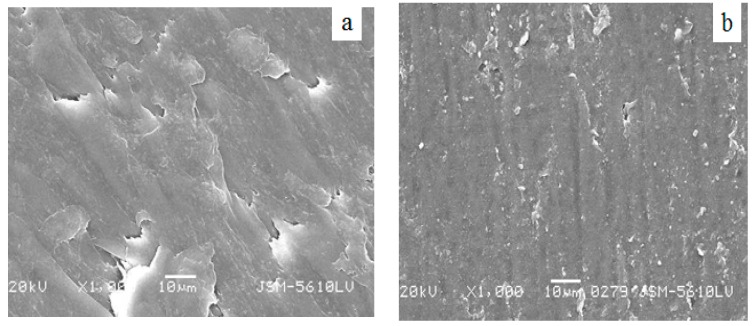
SEM micrographs for the PA66/PVDF and VMg-PA66/PVDF blends. (**a**) PA66/PVDF(40/60); (**b**) 3 wt %VM-*g*-PA66/PVDF(40/60).

Figure 6 is the SEM image of PA66/PVDF blends. Figure 6a is the SEM image of PA66/PVDF. It can be seen from Figure 6a that PA66/PVDF blends present a unique structure of ordered arrangement which is evenly distributed. It can be seen clearly that the particles of PA66 are evenly distributed in PVDF. VM-*g*-PA66 is as the dispersion phase of the main ingredient which is dispersed in flakes, where the continuous phase gives priority to PVDF. With regard to the amplification of the continuous phase, it can also be found that there are small VM-*g*-PA66 particles. All of these are demonstrated that PA/PVDF blends are a two-phase system. Figure 6b is the SEM image of VM-*g*-PA66/PVDF blends after adding the bulking agent of VM. It can be seen from the figure that PA66/PVDF blend film presents a two-phase separation structure in PA66/PVDF blends. PA66 as the dispersion phase of the main ingredient is dispersed in flakes in the continuous phase where it gives priority to PVDF, and the interface adhesion is not good. Figure 6b displays that the accession of 3 wt % bulking agent VM makes the interface of dispersion phase and continuous phase blurred with the melt blending of PA66 and PVDF. This shows that the accession of bulking agent VA-MA can improve interface adhesion between the PVDF phase and PA66 phase.

### 3.2. Dielectric Properties

**Figure 7:**
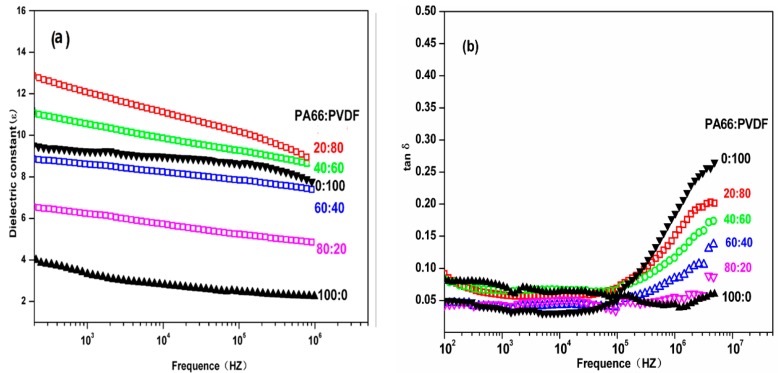
Frequency dependence of (**a**) ε and (**b**) tanδ for PA66/PVDF blends.

Figure 7 is the relationship of dielectric constant ε of PA66/PVDF blend system to frequency. In PA66/PVDF blend system. It can be seen from the figure that the dielectric constant ε of PA/PVDF blend system has no significant change with the increase of electric field frequency, which presents excellent frequency stability. After PA and PVDF are melted and blended according to different volume ratios, the dielectric constants of all the volume ratios of PA66/PVDF blends decrease with the increase of frequency. It can also be seen from the figure that the dielectric loss of PA66/PVDF blends in different volume ratios increases with the increase of frequency after PA66 and PVDF are melted and blended in different volume ratios, but it is slower than the speed of pure PVDF. In the low frequency region (*f* < 10^5^ Hz), the dielectric loss tanδ of PA66/PVDF is less than 0.10, and there is no significant difference among the volume ratios. In the high frequency region (such as 10^6^ Hz), the dielectric loss of PA66/PVDF blends decreases continuously with the increase of PA66 volume content in the blend system. This is because the dielectric loss of PA66 is lower than PVDF in high frequency region, and polar amide groups can promote the orientation of CF_2_ in high frequency, decreasing thermal loss of the blends. This shows that the interaction of PA66 and PVDF requires a better compatibility with the melt blending of PA66 and PVDF.

**Figure 8:**
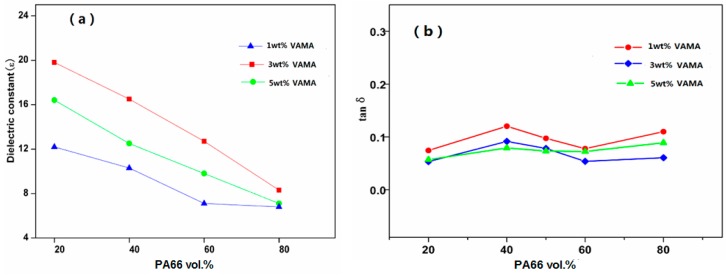
Variation of (**a**) ε and tanδ (**b**) *versus* VM content of VM-*g*-PA66/PVDF blends.

Figure 8 is the melt blending of PA66 and PVDF after VM is added, by which it can not only improve the dielectric constant of PA66/PVDF blends to some extent. Meanwhile, it can reduce the dielectric loss of blends. VA-MA makes polar dipoles easier to be oriented in the PA66/PVDF blend system, thus lowering the dielectric loss of blends.

PA66/PVDF blends present excellent dielectric properties. However, combining the result of thermal property analysis on three PA/PVDF blends in the last section, it can be found that the melting point (*T*_m_ = 223.2 °C) of PA66 is high. During the melt blending with PVDF (*T*_m_ = 163.4 °C), it requires a very high processing temperature. In the temperature rising process, it may destroy the crystal form of the polymer and cause inconvenience for preparation. Therefore, VM is selected to provide graft modification on PA66 to study the influence it has on PVDF blends’ other properties.

At room temperature, when the frequency is 10^3^ Hz, the dielectric properties of VM-*g*-PA66/PVDF blends are obtained for analysis after adding different mass fractions of VM. The quantities of VM added are 1 wt %, 3 wt %, and 5 wt %. The relation curve of the dielectric constants of VM-*g*-PA66/PVDF blends to the content of VM is shown in Figure 8. Besides, the imaginary line means VM-*g*-PA66/PVDF blends of VM which are not added. Obviously, the influence of bulking agent VM-*g*-PA66/PVDF on the dielectric constant of blends was 3 wt % > 1 wt % > 5 wt %. When PA66 and PVDF are melted and blended, it can improve the flexibility of molecules with the accession of VM, and the dielectric constant of VM-*g*-PA66/PVDF can be improved. However, excessive VM can improve the grafting ratio of PA66, and the crystallinity of PA66 is declined, which is not good for the collection and delivery of system charge.

### 3.3. Mechanical Properties

**Figure 9:**
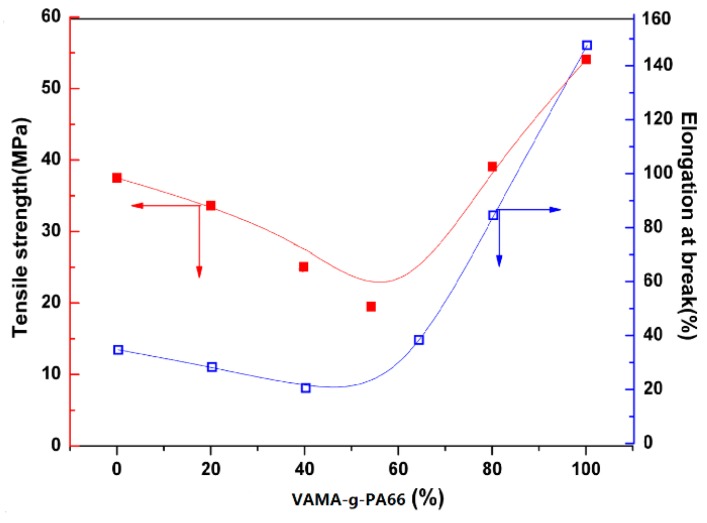
Variations of the tensile strength and the tensile elongation of VM-*g*-PA66/PVDF blends *versus* VM-*g*-PA66 volume fraction.

Figure 9 is the influence curve of PA66 volume content on VM-*g*-PA66/PVDF blend stretching strength and breaking elongation. It can be seen clearly that stretching strength presents irregular variations with the increase of PA66 content, which decreases gradually first. When VM-*g*-PA66 was about 60 vol %, the minimum stretching strength was 16.13 MPa, and then it increased sharply. Meanwhile, breaking elongation is also declining and then rising with the increase of PA66 content. Coincidently, when the volume content of PA66 is 60 vol %, both of the values are the minimum. The elongation of blends is over 20%, and stretching strength was over 18 MPa, which shows that the melting blends of VM-*g*-PA66 and PVDF were suitable to be used for flexible dielectric material.

## 4. Conclusions

This paper demonstrated a simple, efficient, and repeatable route to produce discontinuous all-polymer blends composed of VM-*g*-PA66 and PVDF. The alternating copolymer VM prepared can be grafted on the molecular chain of PA66. VM-*g*-PA66 can influence α crystal orientation and crystal transformation of PVDF. By adding VM at 1 wt %, we can improve the dielectric constant ε of VM-*g*-PA66/PVDF blends without impact on dielectric loss. The all-polymer blends having a relatively high-ε, low dielectric loss, and excellent storage modulus as induced by interfacial copolymer VM. When the quantity of VM added was 3%, the dielectric constant ε of VM-*g*-PA66/PVDF blends declined sharply. The dielectric constant ε of PA66/PVDF blends can be up to 25 after capacity expansion. The elongation of blends was over 20%, stretching strength was over 16.13 MPa. The melted blends of PA66 and PVDF can be used as flexible dielectric material.
